# Modelling collective invasion with reaction–diffusion equations: When does domain curvature matter?

**DOI:** 10.1016/j.aml.2024.109315

**Published:** 2025-02

**Authors:** J.J. Pollacco, R.E. Baker, P.K. Maini

**Affiliations:** a Department of Biochemistry, University of Oxford, Oxford, OX1 3QU, United Kingdom; b Wolfson Centre for Mathematical Biology, Mathematical Institute, University of Oxford, Oxford, OX2 6GG, United Kingdom

**Keywords:** Reaction–diffusion, Cellular invasion, Fisher-KPP, Annulus

## Abstract

Real-world cellular invasion processes often take place in curved geometries. Such problems are frequently simplified in models to neglect the curved geometry in favour of computational simplicity, yet doing so risks inaccuracies in any model-based predictions. To quantify the conditions under which neglecting a curved geometry is justifiable, we explore the dynamics of a system of reaction–diffusion equations (RDEs) on a two-dimensional annular geometry analytically. Defining ϵ as the ratio of the annulus thickness δ and radius $r_{0}$ we derive, through an asymptotic expansion, the conditions under which it is appropriate to ignore the domain curvature for a general system of reaction–diffusion equations. To highlight the consequences of these results, we simulate solutions to the Fisher–Kolmogorov–Petrovsky–Piskunov (Fisher–KPP) model, a paradigm nonlinear RDE typically used to model spatial invasion, on an annular geometry. Thus, we quantify the size of the deviation from an analogous simulation on the rectangle, and how this deviation changes across the width of the annulus. We further characterise the nature of the solutions through numerical simulations for different values of $r_{0}$ and δ. Our results provide insight into when it is appropriate to neglect the domain curvature in studying travelling wave behaviour in RDEs.

## 1 Introduction

Reaction–diffusion models are frequently used in applied mathematics to model invasion processes, finding use in collective cell migration and wound healing [1], [2], [3], [4], tumour growth [5], and in ecology [6]. Invasion processes on curved domains are prolific in nature, and so an emerging trend in both experiments on, and modelling of, such processes is to examine invasion in curved geometries [7], [8], [9], [10]. However, it is often desirable to simplify a calculation or simulation by neglecting the curvature of the domain, for example in modelling of the cranial neural crest, a powerful paradigm of collective cell migration [11]. Therefore, understanding the impact of domain curvature in such models is critical to ensure that the model predictions are accurate.

The starting point for many reaction–diffusion models in two spatial dimensions is the general system of reaction–diffusion equations (RDEs) for a collection of n scalar fields $u\left(x,y,t\right)={\left(u_{1}\left(x,y,t\right),\ldots ,u_{n}\left(x,y,t\right)\right)}^{T}$. For the ith field $u_{i}$,

(1) $$\frac{\partial u_{i}}{\partial t}=\nabla \cdot \left(D_{i}\left(u\right)\nabla u_{i}\right)+F_{i}\left(u\right),$$

where $D_{i}\left(u\right)>0$ and $F_{i}\left(u\right)$ are components of the diffusion and reaction vectors $D\left(u\right)={\left(D_{1}\left(u\right),\ldots ,D_{n}\left(u\right)\right)}^{T}$ and $F\left(u\right)={\left(F_{1}\left(u\right),\ldots ,F_{n}\left(u\right)\right)}^{T}$, respectively. For each component i, we require that Eq. (1) has at least one positive stable steady state in the absence of diffusion. To gain insight into invasion on a curved domain, we first study a generic system of RDEs on an annular domain, exploring the deviation of their behaviour from those on rectangles.

## 2 Results: RDEs on an annular geometry

### 2.1 Asymptotic expansion of an RDE system in an annular geometry

**Fig. 1:**
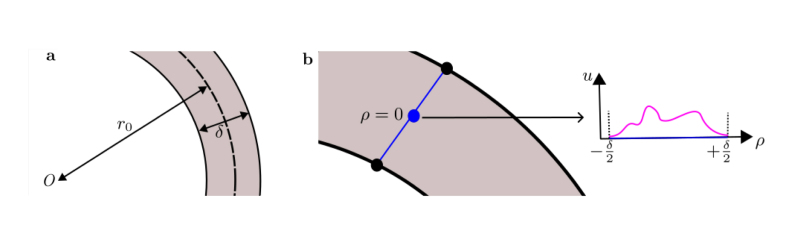
**a** Definition of the annulus thickness δ and its radius $r_{0}$. **b** Demonstration of the solution case we consider in Section 2.1. An arbitrary non-zero solution profile u(ρ,θ,t) (magenta) is considered on the annulus on a line of constant θ (blue) at one time. The solution profile along that line of constant θ is further represented as a function of $\overset{ˆ}{\rho }$ to the right of the annulus.

For an annular geometry, we define its radius $r_{0}$ as the radius of the circle defining all points equidistant from its inner and outer circles, and the thickness δ as the difference between the radii of the outer and inner circles (Fig. 1**a**). We define the parameter $\epsilon =\delta /r_{0}$. In this section we argue that, on the annulus, domain curvature does not substantially affect the solution profile obtained when ϵ is small. We further show that radial dynamics dominate the solution, and that the azimuthal dynamics provide only a subleading correction of $O\left(\epsilon^{2}\right)$. We work in a modified polar coordinate system $\rho =r-r_{0}$ with ρ∈[−δ/2,δ/2] and θ the usual azimuthal angle. Eq. (1) can then be written in ρ and θ coordinates as

(2) $$\frac{\partial u_{i}}{\partial t}=\frac{\partial }{\partial \rho }\left(D_{i}\left(u\right)\frac{\partial u_{i}}{\partial \rho }\right)+\frac{D_{i}\left(u\right)}{r_{0}+\rho }\frac{\partial u_{i}}{\partial \rho }+\frac{1}{{\left(r_{0}+\rho \right)}^{2}}\frac{\partial }{\partial \theta }\left(D_{i}\left(u\right)\frac{\partial u_{i}}{\partial \theta }\right)+F_{i}\left(u\right).$$

Since we are interested in determining the relative contributions of the radial and azimuthal terms in Eq. (2), we first normalise $u_{i}$ over its typical scale of variation $K_{i}$ such that $u_{i}=K_{i}\overset{ˆ}{u_{i}}$, and, assuming they exist and are of similar size between the species, define characteristic diffusion coefficient and reaction scales

$$D_{i}\left(u\right)=\Delta_{i}\overset{ˆ}{d_{i}}\left(\overset{ˆ}{u}\right),\;\;F_{i}\left(u\right)=k_{i}\overset{ˆ}{f_{i}}\left(\overset{ˆ}{u}\right),$$

where $\Delta_{i},k_{i}$ are positive, dimensional constants. We then rescale to choose the timescale of interest, T, to be the characteristic time for diffusion across the thickness of the annulus. We also rescale the radial coordinate on this length scale, giving

$$T_{i}=\delta^{2}\Delta_{i}^{-1},\;\;\overset{¯}{k_{i}}=T_{i}K_{i}^{-1}k_{i},\;\;\overset{ˆ}{\rho }=\rho \delta^{-1},\;\;\overset{ˆ}{t}=T^{-1}t.$$

Writing $u_{i}\left(\rho ,\theta ,t\right)/K_{i}=\overset{ˆ}{u_{i}}\left(\overset{ˆ}{\rho },\theta ,\overset{ˆ}{t}\right)$, we obtain

(3) $$\frac{\partial \overset{ˆ}{u_{i}}}{\partial \overset{ˆ}{t}}=\frac{\partial }{\partial \overset{ˆ}{\rho }}\left(\overset{ˆ}{d_{i}}\left(\overset{ˆ}{u}\right)\frac{\partial \overset{ˆ}{u_{i}}}{\partial \overset{ˆ}{\rho }}\right)+\frac{\overset{ˆ}{d_{i}}\left(\overset{ˆ}{u}\right)}{\overset{ˆ}{\rho }+\frac{r_{0}}{\delta }}\frac{\partial \overset{ˆ}{u_{i}}}{\partial \overset{ˆ}{\rho }}+\frac{1}{{\left(\overset{ˆ}{\rho }+\frac{r_{0}}{\delta }\right)}^{2}}\frac{\partial }{\partial \theta }\left(\overset{ˆ}{d_{i}}\left(\overset{ˆ}{u}\right)\frac{\partial \overset{ˆ}{u_{i}}}{\partial \theta }\right)+\overset{¯}{k}\overset{ˆ}{f_{i}}\left(\overset{ˆ}{u}\right).$$

Rewriting Eq. (3) in terms of ϵ gives

(4) $$\frac{\partial \overset{ˆ}{u_{i}}}{\partial \overset{ˆ}{t}}=\frac{\partial }{\partial \overset{ˆ}{\rho }}\left(\overset{ˆ}{d_{i}}\left(\overset{ˆ}{u}\right)\frac{\partial \overset{ˆ}{u_{i}}}{\partial \overset{ˆ}{\rho }}\right)+\frac{\epsilon \overset{ˆ}{d_{i}}\left(\overset{ˆ}{u}\right)}{1+\epsilon \overset{ˆ}{\rho }}\frac{\partial \overset{ˆ}{u_{i}}}{\partial \overset{ˆ}{\rho }}+\frac{\epsilon^{2}}{{\left(1+\epsilon \overset{ˆ}{\rho }\right)}^{2}}\frac{\partial }{\partial \theta }\left(\overset{ˆ}{d_{i}}\left(\overset{ˆ}{u}\right)\frac{\partial \overset{ˆ}{u_{i}}}{\partial \theta }\right)+\overset{¯}{k}\overset{ˆ}{f_{i}}\left(\overset{ˆ}{u}\right).$$

We assume there is some variation in u in the radial direction (Fig. 1**b**), which to be observable must be over a length scale ∼δ, so that the $\overset{ˆ}{\rho }$ gradients are non-zero. Assuming ϵ to be small, we further asymptotically expand each $\overset{ˆ}{u_{i}}\left(\overset{ˆ}{\rho },\theta ,\overset{ˆ}{t}\right)={\overset{ˆ}{u}}_{i,0}\left(\overset{ˆ}{\rho },\theta ,\overset{ˆ}{t}\right)+\epsilon {\overset{ˆ}{u}}_{i,1}\left(\overset{ˆ}{\rho },\theta ,\overset{ˆ}{t}\right)+\epsilon^{2}{\overset{ˆ}{u}}_{i,2}\left(\overset{ˆ}{\rho },\theta ,\overset{ˆ}{t}\right)+\cdots \;$, and the $\overset{ˆ}{d_{i}}$ and $\overset{ˆ}{f_{i}}$ in terms of the $\overset{ˆ}{u_{i}}$. The dynamics of ${\overset{ˆ}{u}}_{i,0}$, the lowest order contribution to the solution, are then determined by

(5) $$\frac{\partial {\overset{ˆ}{u}}_{i,0}}{\partial \overset{ˆ}{t}}=\frac{\partial }{\partial \overset{ˆ}{\rho }}\left(\overset{ˆ}{d_{i}}\left(\overset{ˆ}{u_{0}}\right)\frac{\partial {\overset{ˆ}{u}}_{i,0}}{\partial \overset{ˆ}{\rho }}\right)+\overset{¯}{k}\overset{ˆ}{f_{i}}\left(\overset{ˆ}{u_{0}}\right).$$

This is a RDE in one spatial dimension, with spatial variation in the radial coordinate $\overset{ˆ}{\rho }$. Thus the dynamics of ${\overset{ˆ}{u}}_{i,0}$, in the radial direction, are the leading order contribution to the solution. It is easy to show that the dynamics of ${\overset{ˆ}{u}}_{i,1}$ are determined again only by terms which depend on gradients in $\overset{ˆ}{\rho }$. Then, ${\overset{ˆ}{u}}_{i,0}$ and ${\overset{ˆ}{u}}_{i,1}$, and zeroth and first order terms from expansion of the other scalar fields (${\overset{ˆ}{u}}_{j,0}$ and ${\overset{ˆ}{u}}_{j,1}$ with j≠i), influence higher order terms, whose temporal evolution is determined by a combination of azimuthal and radial gradients at $O\left(\epsilon^{2}\right)$. Thus, the dominant behaviour of the solution is in the radial direction to O(ϵ). The equations governing the dynamics at O(ϵ) and $O\left(\epsilon^{2}\right)$ are provided in the Supplementary Information.

To demonstrate the consequences of these analytic calculations numerically, we now focus on the paradigm RDE for studying spatial invasion, the single-species Fisher–KPP equation [12], [13]. The Fisher–KPP equation is given by

(6) $$\frac{\partial u}{\partial t}=D\nabla^{2}u+ku\left(1-\frac{u}{K}\right),$$

with D, k and K positive constants. The Fisher–KPP equation is well-studied in one spatial dimension and permits travelling wave solutions with speed $c\geq 2\sqrt{Dk}$ [14], with equality achieved as t→∞ when compactly supported initial data are used [15], [16]. For the Fisher–KPP equation, Eq. (6), the analogous O(1) result from the asymptotic analysis is

(7) $$\frac{\partial {\overset{ˆ}{u}}_{0}}{\partial \overset{ˆ}{t}}=\frac{\partial }{\partial \overset{ˆ}{\rho }}\left(\frac{\partial {\overset{ˆ}{u}}_{0}}{\partial \overset{ˆ}{\rho }}\right)+\overset{¯}{k}K\overset{ˆ}{u_{0}}\left(1-\overset{ˆ}{u_{0}}\right),$$

where we have dropped the index i since there is only one scalar field. The associated non-dimensionalisation is

$$T=\delta^{2}D^{-1},\;\;\overset{¯}{k}=TK^{-1}k,\;\;\rho =\delta \overset{ˆ}{\rho },\;\;t=T\overset{ˆ}{t}.$$

Notice that here, by design, K cancels out of the non-dimensionalisation. As we shall see numerically, for the Fisher–KPP equation, the existence of a spatially homogeneous stable steady state allows the gradients in the radial direction to become zero; if this steady state were not present, then the O(1) dynamics would remain the significant contribution to the solution dynamics. Thus, the O(1) and O(ϵ) equations become identically zero after a short time, allowing the dynamics in the azimuthal direction at $O\left(\epsilon^{2}\right)$ to eventually dominate. The following sections explore numerically the consequences of this result for differing ϵ.

### 2.2 Numerical simulation demonstrates similarity to solutions on the rectangle and a timescale separation between radial and azimuthal dynamics

**Fig. 2:**
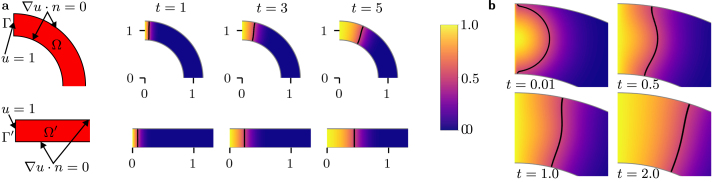
Solution profiles for the Fisher–KPP equation simulated in two dimensions on the rectangle and annulus. Only the upper half of the half-annulus is shown for simplicity. **a** Demonstration of the simulated geometry. Solutions with a Dirichlet boundary condition u=1 on the boundaries $\Gamma ,\Gamma^{\prime }$, and no-flux boundary conditions on the others, evaluated at t=1,3,5. The $u\left(x_{i},y_{i},t\right)=0.5$ isoline, found by selecting coordinates $\left(x_{i},y_{i}\right)$ with u satisfying u∈{|u−0.5|≤0.005}, is indicated by a black line. **b** Solutions with two-dimensional Gaussian initial condition and Dirichlet boundary condition on boundary Γ evaluated at t=0.01,0.50,1.00 and 2.00.

Consider a half-annular domain Ω, with radius $r_{0}$ and thickness δ as above, spanning θ∈[π/2,−π/2]. Taking δ=0.4 and $r_{0}=1$ to set ϵ=0.4, we simulated Eq. (6) on a realisation of Ω generated using Gmsh [17] and by applying the finite element method implemented in FENICSX [18], [19], [20], choosing K=1, D=0.005, k=1 in appropriate units. In the one-dimensional infinite spatial domain case, the width of the wavefront scales as $\left(\sqrt{Dk}\right)$ [14], and so for this parameter choice we anticipated observing the formation of a travelling wave solution in the azimuthal direction with the front localised far from the boundary at θ=−π/2. In the Supplementary Information, we summarise results from equivalent simulations over a range of $k,r_{0}$ and δ. The top vertical edge Γ (see Fig. 2**a**), specified by $\left\{\left(r,\theta \right)\;|\;\theta =\pi /2,r\in \left[r_{0}-\delta /2,r_{0}+\delta /2\right]\right\}$, was supplied with the Dirichlet boundary condition u(r,π/2,t)=1 to mimic a constant solution density entering from outside the domain; Neumann no-flux boundary conditions were imposed on the three other edges. An initial condition u(r,θ,0)=0 for (r,θ)∈Ω/Γ was used. For comparison, we performed equivalent simulations on a rectangle of width δ and length $\pi r_{0}$. We imposed the Dirichlet boundary condition u(0,y,t)=1 on the edge $\Gamma^{\prime }$ specified by {(x,y)|x=0,y∈[0,δ]}.

Fig. 2**a** shows the solutions on the annulus and rectangle, which are qualitatively similar, and for longer times appeared as travelling waves in the θ (annulus) or x (rectangle) coordinates, respectively. Since we observed this close qualitative match, and since our analytic result implies that radial gradients should quickly go to zero, we examined the solution on the annulus for small times in Fig. 2**b** with a radially varying initial condition on Ω. Taking $u\left(x,y,0\right)=\exp\left(-\left(x^{2}+{\left(y-1\right)}^{2}\right)/\alpha^{2}\right),\left\{\left(x,y\right)\notin \Gamma \right\}$ with $\alpha^{2}=0.05$, we found that the radial dynamics dominate the solution at early times. The solution approaches the stable steady state $u^{*}=1$ in the radial direction, and over a longer timescale begins to propagate in the azimuthal direction with little variation of u in the radial coordinate.

### 2.3 Differences between the solution on the rectangle and annulus

**Fig. 3:**
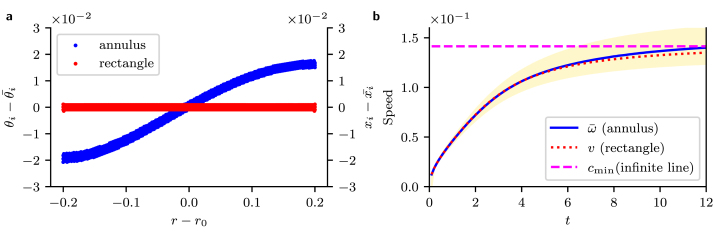
Properties of the u=0.5 isoline in equivalent simulations on the rectangle and annulus at different times. **a** Deviation of $\theta_{i}$ of the isoline positions from the mean angle $\overset{¯}{\theta_{i}}$ in simulations on the annulus, and of the $x_{i}$ from the mean x-coordinate $\overset{¯}{x}$ on the rectangle. Both isolines are displayed at t=2.0. **b** Values of the mean angular speed $\overset{¯}{\omega }=\langle \omega_{i}\left(t\right)\rangle$ of the points on the isoline on the annulus (blue) and linear speed $v=\langle v_{i}\left(t\right)\rangle$ on the annulus. The range of values of $\omega_{i}\left(t\right)$ for the annulus are shown (gold overlay). The minimum speed for a travelling wave solution allowed by Fisher’s equation on an infinite line is also shown (magenta). Note that all points sampled within the tolerance of the isoline are shown, so the profiles appear slightly jagged and thick due to the imperfect alignment between the isoline position and element positions on the mesh.

Despite being qualitatively similar, the solution on the annulus does not perfectly match the solution on the rectangle. To examine to what extent the solution deviates, we investigated the coordinates $\left(x_{i},y_{i}\right)$ and $\left(r_{i},\theta_{i}\right)$ on the u=0.5 isoline on the rectangle and the annulus, respectively. We plotted the angles $\theta_{i}$ (annulus) or x-coordinates $x_{i}$ (rectangle) for points on the isoline (Fig. 3**a**). On the rectangle we found, as expected, that the isoline lies on a line of constant x at any given time, with the solution propagating in the x-direction. For the annulus, the solution propagates azimuthally, similar to the rectangle, but points on the isoline deviated from the mean $\overset{¯}{\theta_{i}}$ of the $\theta_{i}$, with the points at the inner radius being at smaller angles than those at the outer radius. Interestingly, we saw the variation in $\theta_{i}$ was preserved at later times.

We also examined the angular speed $\omega_{i}$ (annulus) and linear speed $v_{i}$ (rectangle) of the points on the isoline (Fig. 3**b**), by calculating their velocity as in [10], giving

$$v_{i}\left(x_{i},y_{i}\right)=\frac{\partial u}{\partial t}\frac{1}{{\left|\nabla u\right|}^{2}}\nabla u=v_{i}\left(r_{i},\theta_{i}\right)⟹\omega_{i}=\frac{v_{i}\left(r_{i},\theta_{i}\right)\cdot {\overset{ˆ}{\theta }}_{i}}{r_{i}},$$

with $\overset{ˆ}{\theta_{i}}=\overset{ˆ}{x}\cos\theta_{i}-\overset{ˆ}{y}\sin\theta_{i}$ the usual polar azimuthal vector in terms of $\overset{ˆ}{x}$, $\overset{ˆ}{y}$, the cartesian unit vectors in two dimensions. The mean speed of both isolines is similar and for later times, both mean speeds approach the theoretical speed for the Fisher–KPP equation in one dimension. On both the rectangle and annulus, it is important to stress that we have not observed the constant speed, fixed profile travelling waves typically used in analysis of the Fisher–KPP equation as we are not in the t→∞ infinite domain limit.

### 2.4 Annulus width and radius determine size of deviation of solutions

**Fig. 4:**
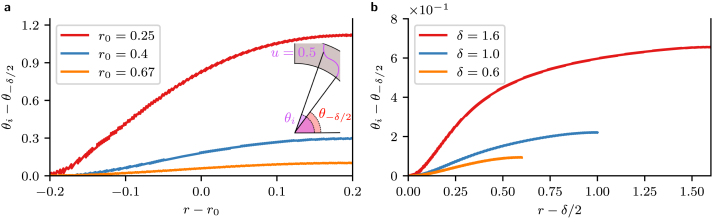
Profiles for the u=0.5 isoline at t=4 for varying radius $r_{0}$ or thickness δ. **a** Simulations utilising different values of $r_{0}=0.25,0.4,0.67$ (ϵ=1.6,1,0.6) with fixed thickness δ=0.4. The difference between $\theta_{i}$ and the angle of the u=0.5 isoline at the inner radius $r_{0}-\delta /2$, $\theta_{-\delta /2}$ over the radial coordinate at t=3.0 is shown. The inset illustrates the definition of $\theta_{-\delta /2}$. **b** Simulations utilising different values of δ=0.6,1.0,1.6 with fixed radius $r_{0}=1$ (ϵ=1.6,1,0.6). The difference between $\theta_{i}$ and the angle of the u=0.5 isoline at the inner radius $\left(r_{0}-\delta /2\right)$, $\theta_{-\delta /2}$, over the radial coordinate at t=3.0, is shown.

As we showed in Section 2.1, radial gradients in the Fisher–KPP equation are on the length scale δ (annulus thickness), whereas derivatives in θ are coupled to r and thus are related to both $r_{0}$ (annulus radius) and δ. To characterise the range of ϵ that may be considered small, we simulated the Fisher–KPP equation with the same initial and boundary conditions as in Section 2.2 but varying either $r_{0}$ or δ independently (Fig. 4). For all cases, the $\theta_{i}$ lagged behind $\theta_{-\delta /2}$ (the angle of the u=0.5 isoline at the inner radius of the annulus). For annuluses with decreasing $r_{0}$ but constant δ, there was an increased spread in the angles of the isoline. Increasing δ with $r_{0}$ constant showed an increasing spread in the $\theta_{i}$ from the inner to outer edge of the annulus. Varying both δ and $r_{0}$ between simulations by the same factor, however, showed only a small difference in the $\theta_{i}$ between simulations (not shown). This provides further evidence that ϵ governs the magnitude of azimuthal variations in the solution. In fact, we found that even for ϵ∼0.6, the solution on the annulus is still close to forming azimuthal wavefronts with lines of constant $\theta_{i}$, analogous to the rectangle. In the Supplementary Information, we perform a more complete characterisation of combinations $r_{0},\delta$ and k where the approximation error is small.

## 3 Discussion

We showed that a general system of autonomous RDEs with characteristic diffusion and reaction scales can be described to lowest order in an annular geometry by considering only a radial version of the same RDE in u, and that the azimuthal dynamics provide a subleading correction. The result is applicable to annuluses with a small ratio ϵ of the annulus thickness to radius. To emphasise the consequences of the result, we have demonstrated that the solutions exhibited by the Fisher–KPP equation on annuluses with a small ratio of thickness to radius do not vary substantially from those on the rectangle. This is counter-intuitive; naively, we might expect that how the solutions differ from the rectangle might be dependent only on the radius $r_{0}$, which affects the tightness of the bend the solution propagates around. However, considering δ→0 returns a one-dimensional Fisher–KPP equation only in θ, in which there is trivially no variation in r. The small ϵ limit thus arises when the annulus approaches a circular arc. We further quantified the deviations between computed numerical solutions on the rectangle and annulus when ϵ is increased, showing that there is spreading in the angle of the travelling wave profile as a function of the radial coordinate.

Our results imply that on annuluses of sufficiently small thickness or large radius, it is justifiable to replace this geometry with a rectangle. Our analytic results show this justification holds for systems of RDEs with autonomous reaction and diffusion terms. Our numerical results (see the Supplementary Information for details) confirm that the approximation error is small over a range of parameters. However, the result relies on the coupling of r to θ in the azimuthal derivatives, and so can break down for non-autonomous reaction–diffusion equations. This is potentially of interest in growing domain problems, which include spatially dependent advection terms, or in systems with externally imposed perturbations that introduce a spatial dependence. In these cases, an external characteristic length scale for the advection or spatial perturbation requires care to be taken in deriving the lowest order contributions. Additionally, the result relies on being able to write down characteristic scales for the diffusion and reaction terms, which may not necessarily hold in, e.g. fast-slow systems. In such systems, we envision that additional care should be taken, with each timescale treated separately and compared. Further, if $D_{i}\left(u\right)<0$ for any u, it has been shown that such negative diffusivities may lead to shock fronts [21]. The presence of shock fronts in θ but not r would also require more careful treatment, since an order-by-order asymptotic expansion is not appropriate.

Here, we have examined a half-annular domain embedded in two spatial dimensions. Extensions to this geometry could be considered, for example motion in tortuous microchannels [22]. In this case, where there are a series of repeated half-annular bends with the same width (analogous to constant δ but varying $r_{0}$), these findings can be iterated for each bend separately. For more general domains that are not annular, the analytic results can still be useful. In this more general construction, one could consider a strip-like domain bounded by two $C_{1}$ curves $r_{1}\left(s_{1}\right)$ and $r_{2}\left(s_{2}\right)$, where $s_{1},s_{2}$ are arc length parameters for each curve. For any simply-connected domain like this, it is possible to construct a curve r(s) and associated set of coordinate vectors $\overset{ˆ}{s},\overset{ˆ}{\rho }$ such that r(s) describes a curve inside the domain, $\overset{ˆ}{\rho }$ describes a direction perpendicular to this curve and also to the curve boundaries. The Riemann mapping theorem [23] then guarantees the existence of a conformal map between this strip-like domain and a half-annulus. This conformal map maps the domain boundaries to the inner and outer radii of a half-annulus, and the curve inside to a circular arc. The thickness, δ, of this half-annulus depends on the exact mapping for a given geometry. For problems with D(u)=constant, since the Laplacian is also conformally variant, the steps of the derivation in Section 2.1 can be performed, and the problem then transformed back to coordinates in the original domain. However, the use of the conformal map does not necessarily preserve the curvature of the wavefront. Instead, the main point that carries over from our results and numerics is that propagation of the solution (for the case of RDEs with travelling wave solutions) obeys a timescale separation if the width of the strip is thin, with propagation of the wave along the length of the strip slower than along its width. For the D(u)≠ constant case, our scale separation may no longer hold. In this case, we expect that the exact nature of the scale separation will depend on the shape of the domain and upon what length scale the two curves which define the domain vary relative to each other. Additionally, our results considered a two-dimensional geometry embedded in two spatial dimensions. However, problems of collective migration on a two-dimensional geometry embedded in three spatial dimensions, and three-dimensional geometries are also of relevance, for example, to fully describe active swimmers in three spatial dimensions [24]. We leave these geometric extensions as an open problem.

Overall, our findings have important implications for simulation of invasion processes on relevant natural geometries. In particular, we see that we can neglect the effect of curvature for a range of situations that may be relevant, for example, in the cranial neural crest, collective cell migration through torturous microchannels and other complex environments [24], [25], or reactions in annular geometries.

## CRediT authorship contribution statement

**J.J. Pollacco:** Contributed to study conceptualisation, performed simulations and analysed the results, wrote and revised the manuscript. **R.E. Baker:** Conceived and supervised the study, wrote and revised the manuscript. **P.K. Maini:** Conceived and supervised the study, wrote and revised the manuscript.

## Appendix A Supplementary data

MMC S1 Supplementary Information, which contains higher order terms in the asymptotic expansion and a numerical exploration of under which parameter ranges the approximation is justified.

A python implementation of the simulations described in the manuscript is available at https://github.com/FusionLocus/fisher-kpp-annulus.
