# Supplementary material for: Modelling collective invasion with reaction–diffusion equations: When does domain curvature matter?
Source: Appl Math Lett. 2025 Feb;160:None. doi: 10.1016/j.aml.2024.109315 (PMC12979725; doi:10.1016/j.aml.2024.109315)
Supplement: MMC S1 — Supplementary Information, which contains higher order terms in the asymptotic expansion and a numerical exploration of under which parameter ranges the approximation is justified. [file mmc1.pdf]

Supplementary Information

S1. Details of the asymptotic expansion for higher order terms

In Sec. 2.1 of the main text, we asserted that the dominant contribution to the dynamics of the solution was due to gradients in the radial direction, up to $\mathcal{O}(\epsilon^2)$. This can be easily shown by grouping in terms of powers of ϵ. Below, we provide the form of the $\mathcal{O}(\epsilon)$ and $\mathcal{O}(\epsilon^2)$ contributions. The $\mathcal{O}(\epsilon)$ contribution is

$$\frac{\partial \hat{u}_{i,1}}{\partial \hat{t}} = \frac{\partial}{\partial \hat{\rho}}\left(\hat{\mathbf{u}}_1 \cdot \left(\left.\frac{\partial \hat{d}_i}{\partial \hat{\mathbf{u}}}\right|_{\hat{\mathbf{u}}=\hat{\mathbf{u}}_0}\right)\frac{\partial \hat{u}_{i,0}}{\partial \hat{\rho}} + \hat{d}(\hat{\mathbf{u}}_0)\frac{\partial \hat{u}_{i,1}}{\partial \hat{\rho}}\right) + \hat{d}(\hat{\mathbf{u}}_0)\frac{\partial \hat{u}_{i,0}}{\partial \hat{\rho}} + \bar{k}\left(\left.\frac{\partial \hat{f}_i}{\partial \hat{\mathbf{u}}}\right|_{\hat{\mathbf{u}}=\hat{\mathbf{u}}_0}\right)\cdot \hat{\mathbf{u}}_1, \quad \text{(S1)}$$

where all dots are taken to be in the space of $\mathbf{u}$. The $\mathcal{O}(\epsilon^2)$ contribution is:

$$\begin{aligned}\frac{\partial \hat{u}_{i,2}}{\partial \hat{t}} = {} & \frac{\partial}{\partial \hat{\rho}}\left(\left(\sum_{j=1}^{N}\hat{u}_{j,1}^2 \left.\frac{\partial^2 \hat{d}_i}{\partial \hat{u}_j^2}\right|_{\hat{\mathbf{u}}=\hat{\mathbf{u}}_0} + \hat{\mathbf{u}}_2 \cdot \left.\frac{\partial \hat{d}_i}{\partial \hat{\mathbf{u}}}\right|_{\hat{\mathbf{u}}=\hat{\mathbf{u}}_0}\right)\frac{\partial \hat{u}_{i,0}}{\partial \hat{\rho}}\right) + \frac{\partial}{\partial \hat{\rho}}\left(\hat{\mathbf{u}}_1 \cdot \left(\left.\frac{\partial \hat{d}_i}{\partial \hat{\mathbf{u}}}\right|_{\hat{\mathbf{u}}=\hat{\mathbf{u}}_0}\right)\frac{\partial \hat{u}_{i,1}}{\partial \hat{\rho}}\right) + \\ & \frac{\partial}{\partial \hat{\rho}}\left(\hat{d}_i(\hat{\mathbf{u}}_0)\frac{\partial \hat{u}_{i,2}}{\partial \hat{\rho}}\right) - \hat{\rho}\hat{d}_i(\hat{\mathbf{u}}_0)\frac{\partial \hat{u}_{i,0}}{\partial \hat{\rho}} + \hat{\mathbf{u}}_1 \cdot \left(\left.\frac{\partial \hat{d}_i}{\partial \hat{\mathbf{u}}}\right|_{\hat{\mathbf{u}}=\hat{\mathbf{u}}_0}\right)\frac{\partial \hat{u}_{i,0}}{\partial \hat{\rho}} + \hat{d}_i(\hat{\mathbf{u}}_0)\frac{\partial \hat{u}_{i,1}}{\partial \hat{\rho}} + \\ & \frac{\partial}{\partial \theta}\left(\hat{d}_i(\hat{\mathbf{u}}_0)\frac{\partial \hat{u}_{i,0}}{\partial \theta}\right) + \left(\sum_{j=1}^{N}\hat{u}_{j,1}^2 \left.\frac{\partial^2 \hat{f}_i}{\partial \hat{u}_j^2}\right|_{\hat{\mathbf{u}}=\hat{\mathbf{u}}_0} + \hat{\mathbf{u}}_2 \cdot \left.\frac{\partial \hat{f}_i}{\partial \hat{\mathbf{u}}}\right|_{\hat{\mathbf{u}}=\hat{\mathbf{u}}_0}\right). \quad \text{(S2)}\end{aligned}$$

For the Fisher-KPP equation, Eq. (S1) and Eq. (S2) reduce straightforwardly to

$$\frac{\partial \hat{u}_1}{\partial \hat{t}} = \frac{\partial^2 \hat{u}_1}{\partial \hat{\rho}^2} + \frac{\partial \hat{u}_0}{\partial \hat{\rho}} + \bar{k}\hat{u}_1(1 - 2\hat{u}_0), \quad \text{(S3)}$$

$$\frac{\partial \hat{u}_2}{\partial \hat{t}} = \frac{\partial^2 \hat{u}_2}{\partial \hat{\rho}^2} - \hat{\rho}\frac{\partial \hat{u}_0}{\partial \hat{\rho}} + \frac{\partial \hat{u}_1}{\partial \hat{\rho}} + \frac{\partial^2 \hat{u}_{i,0}}{\partial \theta^2} - 2\bar{k}\hat{u}_1^2 + \bar{k}\hat{u}_2(1 - 2\hat{u}_0). \quad \text{(S4)}$$

S2. Numerical exploration of the approximation error for different parameter values

In Sec. 2.2 in the main text, we demonstrated for a specific set of parameters that the qualitative difference between solutions on the rectangle (that is, a travelling wave profile in the x direction with no variation in the y direction) and those on the annulus (a travelling wave profile in the $\hat{\theta}$ direction with little variation in the $\hat{\mathbf{r}}$ direction) is small relative to the extent of the domain. Although the asymptotic analysis implies that this variation should remain small whilst ϵ is small, it is reasonable to ask over what range of ϵ the result is still valid. Therefore, in this section we provide a numerical exploration of the dynamics for a range of values of the parameters δ, r_0, and $\bar{k}$, the three salient parameters in the Fisher-KPP equation after non-dimensionalisation.

In order to fairly compare the solutions between the annulus and the rectangle, we define the following metrics that summarise the behaviour of the $u = 0.5$ isoline:

1. *Planar deviation:* The quantity $S_1 = \sqrt{\frac{1}{N}\sum_i^N \left(\frac{\nabla u(r_i,\theta_i)}{|\nabla u(r_i,\theta_i)|}\cdot \hat{\mathbf{r}}\right)^2}$, which describes the root mean square value of the dot product of points on the isoline with the radial unit vector. For a wavefront which perfectly approximates the flat wavefront on the rectangle, the value of this metric should be zero since the dot product will be zero. Therefore, this metric measures the deviation of the wavefront from being planar.
2. *Angular spread*: The value of $S_2 = |\theta_{\delta/2} - \theta_{-\delta/2}|$. This directly measures how much the wavefront spreads over the radial direction.
3. *Speed deviation*: The quantity $S_3 = |\langle\omega_i\rangle - \omega_{r_0}|/\omega_{r_0}$, where ω_{r_0} is the angular speed of the solution on a semi-circular arc of radius r_0, computed from a separate simulation. This constitutes a comparison with the $\epsilon = 0$ limit, since $\delta = 0$. In this case, all the radial gradients are zero by definition, and it is trivial to show that the Fisher-KPP equation is now solved over one spatial dimension, and so has an asymptotic minimum angular speed $2\sqrt{Dk/r_0^2}$. Therefore, this is a direct comparison between the angular speed on the circle which describes a one-dimensional planar front in θ, and on the annulus.

Combined, these metrics describe whether the angular extent, planar nature, and speed of the wavefront are preserved. For the following numerical exploration, we evaluate the metrics at $t = 2.5$. We chose this t as a compromise, with the goal of summarising a wide range of parameter values in mind. For large enough k or ϵ, propagation of the wavefront is too fast, and so is affected by the boundary condition on the lower edge of the half annulus $\theta = -\pi/2$. This would affect our evaluation of the metrics over a significant number of simulations at later t. For smaller times $t = 1, 1.5, 2$, the simulation results yield the same qualitative result whilst attempting to minimise the effect of transient contributions to the wavefront shape or extent.

S2.1. Approximation error as a function of r_0 and $\bar{k}$

We quantify the effect of a larger magnitude reaction term to the approximation as a function of ϵ. However, as we showed through non-dimensionalisation, the value of δ affects the scaling of the reaction term's contribution, and so we choose to vary r_0 to change ϵ, and k to change $\bar{k}$, with constant $\delta = 0.4$, and $D = 0.005$, as in the text. The results are shown in Fig. S1.

For the metrics S_1 and S_2, there is a lower deviation from a planar wavefront for smaller values of $\bar{k}$ and larger r_0 (smaller ϵ). The simulations cover four orders of magnitude for $\bar{k}$. However, interestingly, we see that the range of ϵ for which the approximation error is lower decreases as $\bar{k}$ becomes larger. This range of parameter values uses $k/D \sim 10^5$, and so although the azimuthal dynamics are small relative to the size of the wavefront when $\epsilon \lesssim 0.5$, there is still a visible effect on the shape of the wavefront. Even in this regime, S_3 demonstrates that despite having a curved profile, the wave speeds are still similar between the $\epsilon \to 0$ limit and the finite ϵ regime, with the highest deviation of 0.29 for the smallest r_0 and largest $\bar{k}$ tested. This is a 30% error, but for most of the other simulations shown,

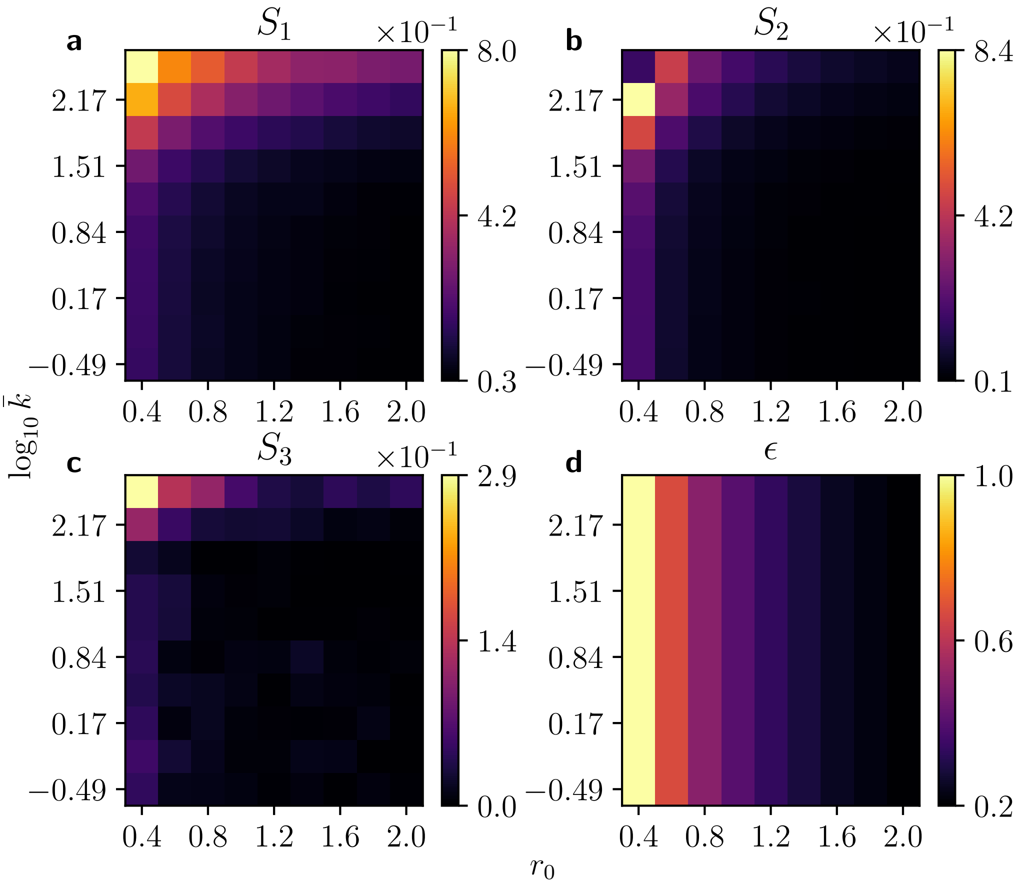

Figure S1: Summary metrics as a function of r_0 and $\bar{k}$. $\bar{k}$ is plotted on a logarithmic scale in each case, as it is varied from $\bar{k} = 0.32, \ldots, 320$. **a** Planar deviation (S_1). **b** Angular spread (S_2). **c** Speed deviation (S_3). **d** Reference ϵ values to aid comparison. Heatmaps are scaled to the maximum and minimum values of these metrics.

the error is below 5%, suggesting that only for $k \gg D$ and small r_0 that substantial errors are incurred.

We note that, for the simulation with $(r = 0.40, \log_{10}\bar{k} = 2.51$ (3 s.f.)), S_2 shows a smaller deviation. Inspection of this simulation showed that 'dip filling' had begun near the lower boundary of the annulus $\theta = -\pi/2$ by $t = 2.5$ for this parameter combination, leading to a smaller spread in the isoline angle in this case.

S2.2. Approximation error as a function of r_0 and δ

In the main text, we demonstrated that there was a qualitative trend in the angular deviation of the $u = 0.5$ isoline as r_0 and δ were varied, to increase the value of ϵ. However, the quantitative spread in the angular deviation is of different magnitude between these cases. Therefore, we quantify the effect of varying each of these parameters independently on the deviation from the planar wavefront solutions on the circle (see Fig S2).

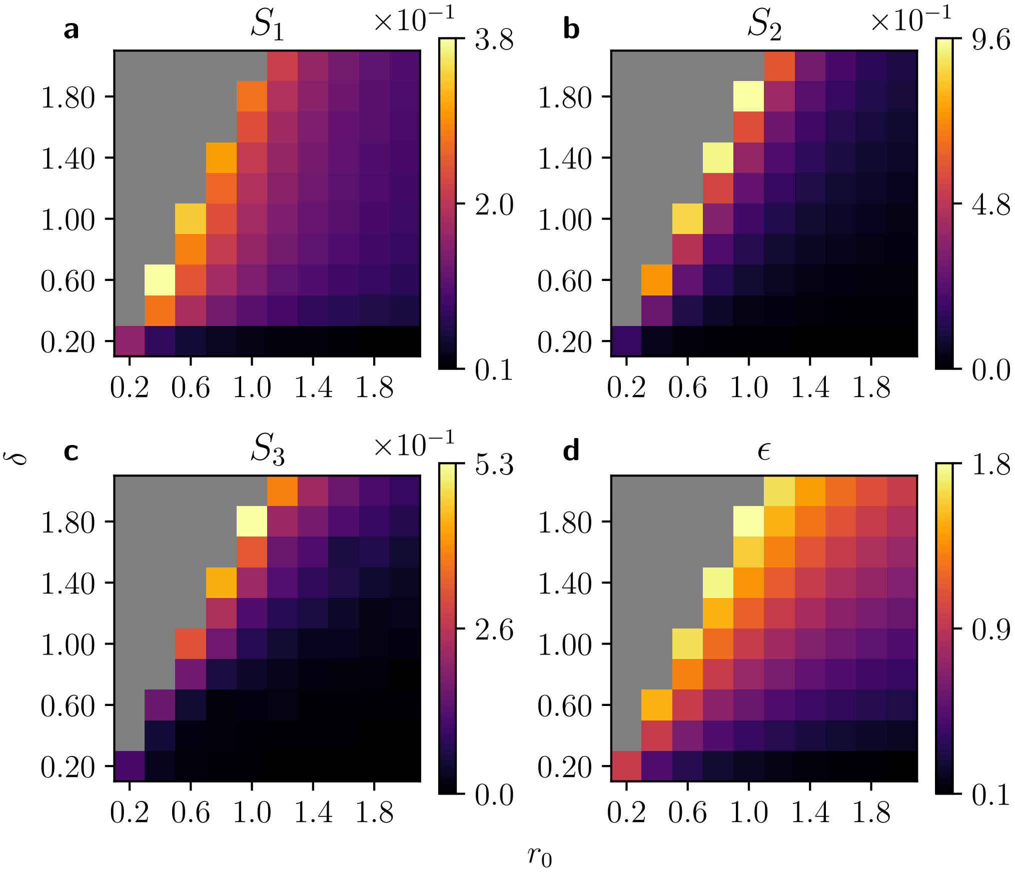

Figure S2: Summary metrics S_1, S_2 and S_3 as a function of δ and r_0. **a** Planar deviation (S_1). **b** Angular spread (S_2). **c** Speed deviation (S_3). **d** Reference ϵ values to aid comparison. Heatmaps are scaled to the maximum and minimum values of these metrics. For $\delta \geq 2r_0$, an annular geometry is no longer formed, and so no simulations exist for this case (coloured grey).

For all metrics, the approximation error decreases with increasing r_0 and increases with increasing δ, as demonstrated in the main text. For S_1, for each r_0 this error increases as ϵ increases towards 2 (as δ approaches $2r_0$), and the wavefronts gain a more curved profile. S_2 also increases for larger values of δ (and thus ϵ), showing that the wavefront spreads out despite being mostly flat. Due to the larger radii, smaller angles are subtended as r_0 increases, but the distance travelled between the isoline at the inner and outer edges of the annulus scales linearly with δ (not shown). Finally, S_3 shows the deviation in angular speed is largest for large ϵ.

S3. Simulation methods

Meshes were produced using Gmsh's python interface, implementing Delaunay triangulation with the characteristic length parameter l_c, which controls the length scale of elements near to the vertices defined at the corners of the mesh. For the rectangular and annulus meshes

used for Sec. 2.2 and Sec. 2.3, $l_c = 0.001$, for the annuluses in Sec. 2.4, $l_c = 0.0025$, and for the numerical exploration in the SI, the annulus meshes used $l_c = 0.005$ and the arc meshes $l_c = 0.0005$. We provide the number of elements in each simulation in Fig. S1 in Tab. S1, and Fig. S2 in Tab. S2. All spatial simulations were performed using the finite element method, implemented in FENICSX using a Lagrange space with continuous elements of order one; measures and calculations involving the gradient of u interpolated the solution and its derivatives into a discontinuous Lagrange space of order zero. Time stepping was implemented using a Crank-Nicolson discretisation with timestep $\delta t = 0.01$, in which the average of the reaction and diffusion terms between two timesteps were taken to represent their values at the intermediate timestep.

S3.1. Number of elements for initial simulations in the main text.

Number of elements for simulation on the base rectangle (width $= \pi$, height $= 0.4$): 2910928.
Number of elements used for simulation on the base annulus ($r_0 = 1$, $\delta = 0.4$): 2912289.
Number of elements used for simulation of annuluses with varying r_0 ($r_0 = 0.25, 0.4, 0.67$, $\delta = 0.4$): 118165, 187779, 313049.
Number of elements used for simulations of annuluses with varying δ ($r_0 = 1$, $\delta = 0.6, 1, 1.6$): 707175, 1167593, 1864381

r_0	Number of elements in arc	Number of elements in annulus
0.4	2518	47611
0.6	3774	71333
0.8	5032	94981
1.0	6288	118125
1.2	7544	141855
1.4	8802	165507
1.6	10058	189105
1.8	11314	212779
2.0	12572	236581

Table S1: Number of elements used in each simulation for the numerical exploration with varying $\bar{k}$ and r_0 values in Sec. S2.1.

r_0 ; δ	0	0.2	0.4	0.6	0.8	1.0	1.2
0.2	252	12305	-	-	-	-	-
0.4	504	24329	47611	71179	-	-	-
0.6	756	36065	71333	106391	141435	176991	-
0.8	1008	48007	94981	141559	187779	235461	280973
1.0	1260	60053	118125	176659	234815	294015	351415
1.2	1508	72057	141891	211985	281733	351883	421735
1.4	1760	84061	165543	247261	328761	400519	491987
1.6	2012	96161	189105	281781	375325	468235	562235
1.8	2264	107597	212775	317085	422639	526651	630723
2.0	2516	119871	236581	352657	468297	585549	701075

r_0 ; δ	1.4	1.6	1.8	2.0
0.2	-	-	-	-
0.4	-	-	-	-
0.6	-	-	-	-
0.8	329809	-	-	-
1.0	410705	467913	528913	-
1.2	492089	561581	632127	701475
1.4	572557	655209	735797	816847
1.6	654491	747995	840687	933481
1.8	736487	841403	946015	1050527
2.0	818525	933695	1051281	1167593

Table S2: Number of elements used in each simulation in the numerical exploration of varying δ and r_0 values. Due to the length of the entries, the table is continued over multiple lines.
